# Triglyceride-glucose index in the prediction of acute kidney injury in patients undergoing coronary artery bypass surgery

**DOI:** 10.3389/fcvm.2025.1572096

**Published:** 2025-05-09

**Authors:** Chen Li, Xingping Lv, Yezhou Shen, Wei Zhou, Tuo Shen, Guoliang Fan, Feng Zhu

**Affiliations:** Department of Critical Care Medicine, Shanghai East Hospital, School of Medicine, Tongji University, Shanghai, China

**Keywords:** triglyceride-glucose, metabolism, coronary artery bypass grafting (CABG), acute kidney injury (AKI), insulin resistance

## Abstract

**Background:**

The triglyceride-glucose (TyG) index, indicative of insulin resistance, is recognized for predicting cardiovascular disease and metabolic disorders, notably kidney disease. In coronary artery bypass grafting (CABG) surgery, its association with postoperative renal injury is significant, suggesting its potential as a predictor for acute kidney injury (AKI) in these patients.

**Methods:**

This single-center, retrospective study included 296 patients. Patients were divided into AKI and non-AKI groups postoperatively according to the KDIGO grading criteria. Multiple linear regression was employed to identify factors influencing the TyG index. Logistic regression was utilized to examine the TyG index's association with AKI in CABG patients. The TyG index's predictive power for postoperative AKI was assessed using receiver operating characteristic (ROC) curve analysis. Assessment of the Predictive Performance of the Prediction Model via Calibration plot and Clinical Decision Curve Analysis.

**Results:**

In comparison between the AKI group and the non- AKI group post-CABG surgery, there was statistically significant differences in TyG index [7.53 [7.25, 7.95] vs. 6.99 [6.64, 7.39], *P* < 0.05]. Logistic regression analysis indicated that for each unit increase in the TyG index, the odds of developing acute kidney injury post-CABG surgery increased by 30.573 times [odds ratio (OR) = 30.573, 95% confidence interval (CI) 3.930–237.807, *P* < 0.001]. The area under the curve (AUC) for the TyG index in predicting postoperative AKI in CABG patients was 0.802 (*P* < 0.001; 95% CI: 0.753–0.851). The calibration plot of the model closely approximated the ideal diagonal line, and the clinical decision curve analysis demonstrated favorable clinical applicability.

**Conclusion:**

Elevated levels of the TyG index are closely associated with the occurrence of AKI in patients following CABG surgery, and the TyG index is a potential indicator for the development of AKI post-CABG.

## 1 Introduction

Coronary artery bypass grafting (CABG) is an effective treatment method for patients with coronary heart disease (1). However, according to literature reports, approximately one-third of patients undergoing CABG surgery experience acute kidney injury (AKI) (2). Currently, clinical assessments of renal function primarily rely on creatinine levels, estimated glomerular filtration rate (eGFR), and other biochemical indicators, but these markers are not capable of providing timely warnings for the onset of AKI (3).

In patients with coronary heart disease, insulin resistance is quite common and is closely associated with chronic kidney disease (4). Insulin resistance can lead to elevated blood glucose levels and increased serum triglyceride levels. Recent research has identified the triglyceride-glucose (TyG) index, which is derived from the logarithmic transformation of the product of plasma triglycerides and fasting blood glucose, as a potential marker for insulin resistance (5, 6). Studies have shown that an increase in the TyG index is closely related to the occurrence and progression of coronary atherosclerosis, coronary heart disease, diabetic nephropathy, and renal microvascular damage (7–11).

The TyG index has been proven to be a reliable indicator of poor prognosis in patients with kidney disease. A close association with elevated TyG index has also been found in cases of contrast-induced nephropathy (12). However, there are very few reports on the relationship between the TyG index and AKI following coronary artery bypass grafting. Therefore, the purpose of this study is to explore the correlation between the TyG index and AKI after CABG surgery, with the aim of providing a reference for the early prevention, monitoring, and treatment of postoperative AKI.

## 2 Methods

### 2.1 Study population

**Figure 1:**
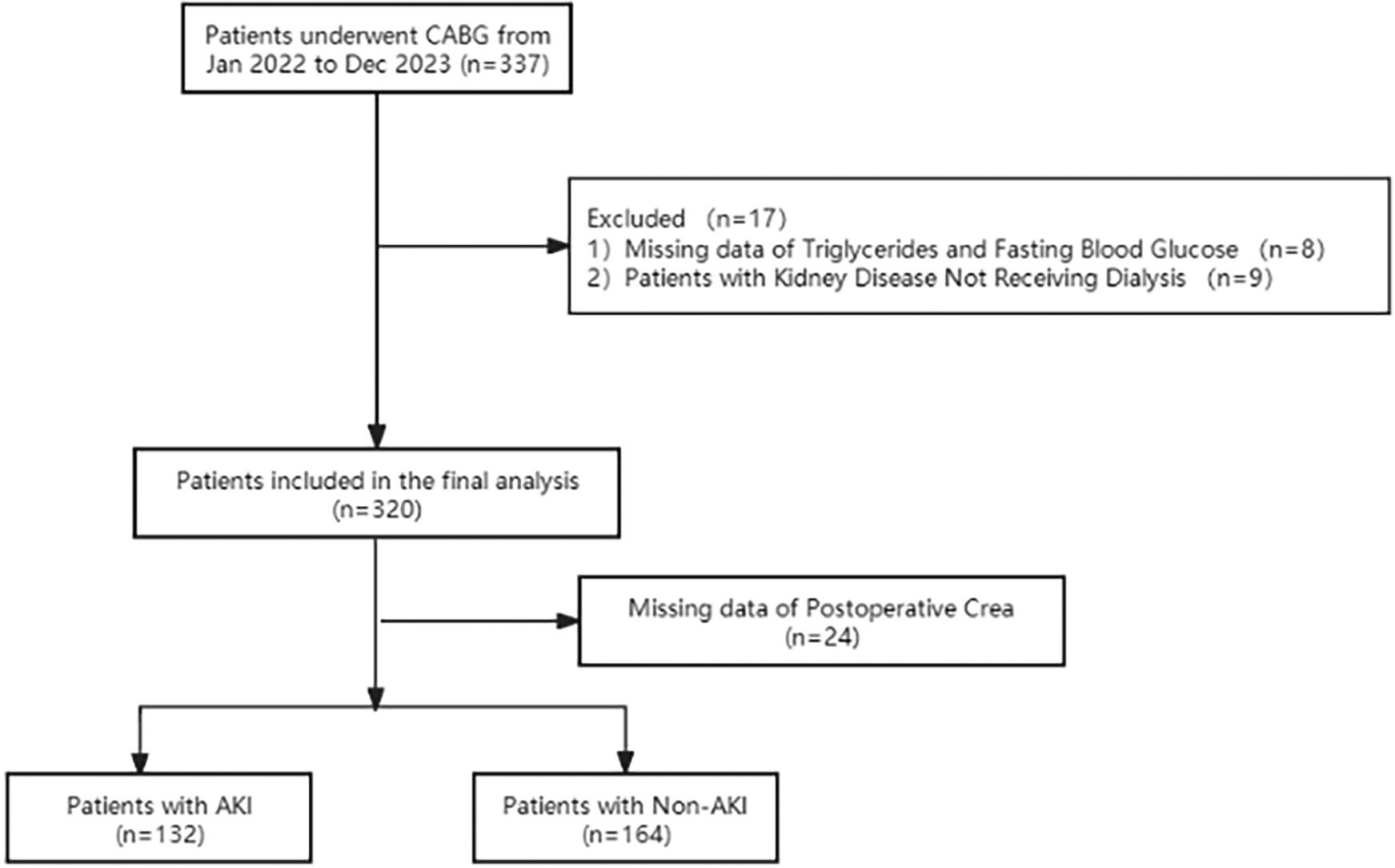
Flow diagram of patient selection.

A total of 296 patients who underwent off-pump CABG surgery at the Shanghai East hospital from January 2022 to December 2023 were included in this study. The inclusion criteria were as follows: 1. Age ≥ 18 years old with complete clinical data; 2. Underwent off-pump CABG surgery after admission. The exclusion criteria were: patients with chronic kidney disease undergoing peritoneal dialysis or hemodialysis, emergency surgery, and those with missing significant clinical data (Figure 1).

### 2.2 Outcomes

Based on the occurrence of AKI following surgery, participants were divided into two groups, namely the AKI group (*n* = 132) and the non-AKI group (*n* = 164). The preoperative, postoperative clinical data were compared. AKI was defined according to the newest consensus-based KDIGO criteria as follows: small changes in serum creatinine (≥0.3 mg/dl or 26.5 mmol/L) when they occurred within 48 h or a maximal change in serum creatinine ≥1.5 times the baseline value until postoperative day 7 compared with preoperative baseline values or urine volume <0.5 ml/kg/h for 6 h (13).

### 2.3 Ethical statement

Approval for this study was obtained from the Committee on Ethics of Biomedical Research at Shanghai East Hospital, Tongji University School of Medicine, Shanghai (No. 2024-YS-043), with a waiver for individual patient consent granted. This study was conducted in accordance with the Declaration of Helsinki (as revised in 2013). Given the observational nature of the study, informed consent of individual patient was waived by the Committee on Ethics at Shanghai East Hospital.

### 2.4 Data collection

Clinical data were collected from the electronic medical record system. The data included patients' general information (gender, age, height, weight), past medical history (hypertension, diabetes, coronary heart disease, heart function classification, atrial fibrillation, hyperlipidemia, myocardial infarction, chronic pulmonary disease, and chronic kidney disease), and clinical data [preoperative white blood cell count (WBC), neutrophil percentage, hemoglobin, platelets, total bilirubin, albumin, creatinine, eGFR, fasting triglycerides, fasting blood glucose, left atrial diameter, left ventricular end-diastolic diameter, left ventricular ejection fraction (LVEF), and EuroSCORE). Body Mass Index (BMI) was calculated as weight divided by the square of the patient's height, expressed in kg/m^2^. The measurements of triglycerides and glucose were completed under fasting conditions preoperatively. The TyG index was calculated as follows: TyG index = ln [fasting triglycerides (mg/dl)] × fasting blood glucose [mg/dl]/2).

### 2.5 Statistical analysis

This study utilized SPSS version 26.0 for statistical analysis. The Shapiro–Wilk test was employed to assess the normality of the data. Continuous data that were normally distributed are presented as the mean ± standard deviation, and the independent samples t-test was used to compare the means between the two groups. Continuous data not normally distributed are expressed as the median M (Q25, Q75), and the Mann–Whitney *U* test was used to compare the medians between the two groups. Categorical data are represented as frequencies and percentages, and the chi-square test or Fisher's exact test was used to assess differences between groups. Pearson's correlation analysis was used to describe the association between variables and the TyG index. Logistic regression analysis was performed to identify the factors influencing the occurrence of acute kidney injury in patients after CABG surgery. The receiver operating characteristic (ROC) curve was used to determine the value of the TyG index in predicting the onset of acute kidney injury after CABG surgery, with the cutoff value derived from the Youden's index (sensitivity + specificity—1). All tests were two-tailed, *P* < 0.05 was considered statistically significant. Subgroup analyses were performed based on age, gender, hypertension, diabetes, hyperlipidemia, myocardial infarction, chronic pulmonary disease, atrial fibrillation and chronic kidney disease to assess the prognostic utility of the TyG index across various patient subgroups. These analyses entailed stratifying patients and evaluating the performance of the TyG index in different groups to identify variations in predictive efficacy. Calibration plot were employed to evaluate the calibration performance of the model. Clinical decision curve analysis (DCA) was conducted to assess its clinical applicability.

## 3 Results

### 3.1 Baseline characteristics

This study included a total of 296 patients who underwent CABG surgery. The baseline clinical characteristics and laboratory measurements of the patients in each group are shown in Table 1. Based on the occurrence of AKI following CABG, the patients were divided into two groups. Patients were stratified into acute kidney injury (AKI) groups according to serum creatinine levels, in alignment with the KDIGO clinical guidelines. There were no statistically significant differences between the groups in terms of gender, age, New York Heart Association (NYHA) classification, coronary heart disease, atrial fibrillation, hyperlipidemia, myocardial infarction, chronic pulmonary disease, preoperative neutrophil percentage, preoperative hemoglobin, preoperative platelet count, preoperative total bilirubin, preoperative albumin, preoperative left ventricular end-diastolic diameter, and preoperative creatinine levels (*P* > 0.05). However, there were statistically significant differences in BMI, hypertension, diabetes, chronic kidney disease, EuroSCORE, preoperative WBC, preoperative left atrial diameter, preoperative LVEF, preoperative fasting triglyceride levels, preoperative fasting blood glucose, TyG index, and preoperative eGFR levels (*P* < 0.05, as shown in Table 1).

**Table 1:** Comparison of preoperative baseline data and preoperative clinical indicators between two groups.

Overall population			
Variable	Non-AKI Group (*n* = 164)	AKI Group (*n* = 132)	*P* value
Male, *n* (%)	133 (81.1)	98 (74.2)	0.161
Age (years)	67.00 [59.00, 73.00]	68.00 [60.00, 75.00]	0.304
BMI (kg/m^2^)	23.80 [22.00, 25.70]	24.73 [22.68, 26.75]	0.027
NYHA, *n* (%)			0.952
I	30 (22.6)	21 (19.6)	
II	64 (48.1)	54 (50.5)	
III	37 (27.8)	30 (28)	
IV	2 (1.5)	2 (1.9)	
Hypertension, *n* (%)	104 (63.41)	99 (75)	0.032
Diabetes mellitus, *n* (%)	59 (35.97)	67 (50.75)	0.013
CAD, *n* (%)	160 (97.56)	126 (95.45)	0.350
AF, *n* (%)	7 (4.3)	5 (3.8)	1.000
Hyperlipidemia, *n* (%)	15 (9.1)	9 (6.8)	0.526
MI, *n* (%)	15 (9.1)	18 (13.6)	0.266
Chronic lung disease, *n* (%)	16 (9.8)	6 (4.5)	0.118
Chronic kidney disease, *n* (%)	2 (1.2)	12 (9.1)	0.002
Euroscore	5.00 [3.00, 6.00]	5.00 [4.00, 7.00]	0.033
White blood cell count (x10^9^/L)	6.26 [5.30, 7.53]	6.75 [5.31, 8.18]	0.020
Neutrophil percentage (%)	61.70 [56.25, 67.50]	61.30 [53.40, 67.50]	0.674
Hemoglobin (g/L)	131.00 [119.00, 138.00]	127.00 [113.00, 138.00]	0.201
Platelet (x10^9^/L)	199.00 [166.00, 236.50]	199.00 [162.00, 238.00]	0.943
TBil (*μ*mol/L)	11.10 [7.95, 14.65]	10.10 [7.60, 13.60]	0.185
ALB (mg/L)	38.74 [37.11, 41.43]	39.10 [37.02, 41.45]	0.644
LAD (mm)	37.00 [34.00, 41.00]	39.00 [36.00, 42.00]	0.005
LVEDD (mm)	47.00 [44.00, 50.00]	48.00 [44.00, 52.00]	0.265
LVEF (%)	63.00 [54.50, 67.00]	60.00 [49.00, 65.00]	0.006
TG (mmol/L)	1.17 [0.97, 1.60]	1.68 [1.37, 2.42]	<0.001
FBG (mmol/L)	5.40 [4.74, 6.30]	6.50 [5.10, 8.61]	<0.001
TyG Index	6.99 [6.64, 7.39]	7.53 [7.25, 7.95]	<0.001
Crea (μmol/L)	72.00 [70.50, 88.00]	101.90 [91.25, 117.50]	0.187
eGFR (ml/min)	87.00 [75.00, 105.00]	69.46 [51.00, 76.25]	0.014

Data are expressed as the median (25th–75th percentiles), or number (percentage). BMI, body mass index; CAD, coronary heart disease; AF, atrial fibrillation; MI, myocardial infarction; TBiL, total bilirubin; Alb,albumin; LAD, left atrial diameter; LVEDD, left ventricular end-diastolic diameter; LVEF, left ventricular ejection fraction; TG, triglyceride; FBG, fasting blood-glucose; TyG index, triglyceride-glucose index; Crea, creatinine; eGFR, estimated glomerular filtration rate.

The postoperative laboratory measurements for patients in each group are presented in Table 2. There were no statistically significant differences between the groups in terms of mechanical ventilation time, total hospital stay, the number of bypass grafts, operative time and the eGFR levels on the day of surgery (*P* > 0.05). However, statistically significant differences were observed when comparing ICU stay duration, creatinine levels on the day of surgery, creatinine and eGFR levels on the first postoperative day, creatinine and eGFR levels on the second postoperative day, and creatinine and eGFR levels on the third postoperative day (*P* < 0.05, as shown in Table 2).

**Table 2:** Comparison of postoperative clinical indicators between two groups.

Overall population			
**Variable**	Non-AKI Group (*n* = 164)	AKI Group (*n* = 132)	*P* value
Number of grafts, *n* (%)			0.152
I	38 (23.2)	22 (16.7)	
II	120 (73.2)	109 (82.6)	
III	6 (3.7)	1 (0.8)	
Operative time (minutes)	200.00 [168.75, 252.50]	210.00 [150.00, 240.00]	0.562
ICU length of stay (minutes)	1404.00 [1238.00, 2745.00]	1693.00 [1371.75, 2940.50]	0.002
Mechanical ventilation (minutes)	317.00 [224.00, 487.00]	347.00 [249.00, 569.75]	0.140
hospital length of stay (days)	15.00 [13.00, 19.00]	16.00 [14.00, 19.25]	0.083
Crea (D1) (μmol/L)	88.50[68.00,104.75]	96.00 [79.25, 163.25]	<0.001
eGFR (D1) (ml/min)	76.50 [64.00, 110.26]	58.50 [36.25, 89.25]	<0.001
Crea (D2) (μmol/L)	82.00 [61.75, 101.25]	111.50 [76.75, 162.75]	<0.001
eGFR (D2) (ml/min)	79.00 [54.25, 115.25]	57.00 [32.00, 88.50]	0.014
Crea (D3) (μmol/L)	74.50 [68.50, 96.60]	104.50 [77.50, 148.00]	<0.001
eGFR (D3) (ml/min)	95.56 [70.00, 111.00]	52.50 [37.25, 86.25]	0.015

Data are expressed as the median (25th–75th percentiles). D0, the day after surgery; D1, the first day after surgery; D2, the second day after surgery; D3 the third day after surgery; Crea, creatinine; eGFR, estimated glomerular filtration rate.

### 3.2 Correlation between TyG Index and baseline data

Pearson's or Spearman's rank correlation analysis was performed to explore the relationship between TyG index and clinical baseline data. As presented in Table 3, the TyG index was positively correlated with Hypertension as well as Diabetes mellitus, BMI, Chronic kidney disease, ICU length of stay, and Preoperative WBC levels and negatively correlated with Preoperative eGFR and LVEF (all *P* < 0.05).

**Table 3:** Correlation between TyG index and potential risk factors.

Variable	Correlation coefficient (r)	*P* value
Hypertension	0.127	0.029
Diabetes mellitus	0.282	<0.001
BMI	0.143	0.014
LVEF	−0.176	0.003
Chronic kidney disease	0.047	0.423
EuroSCORE	−0.028	0.629
Preoperative WBC	0.127	0.029
Preoperative LAD	0.017	0.773
Preoperative eGFR	−0.115	0.024
ICU length of stay	0.149	0.010

BMI, body mass index; LVEF, left ventricular ejection fraction; WBC White blood cell count; LAD, left atrial diameter; eGFR, estimated glomerular filtration rate.

### 3.3 Risk factors for AKI after CABG

Logistic regression analysis revealed that ICU stay duration [OR = 1, 95% CI (1.000, 1.001), *P* = 0.036], and the TyG index [OR = 30.573, 95% CI (3.930, 237.807), *P* < 0.001] are risk factors for the development of acute kidney injury in patients after CABG surgery (*P* < 0.05, see Table 4).

**Table 4:** Logistic regression analysis of AKI in patients.

Variable	*β*	SE	Wald *X*^2^ value	*P* value	OR value	95% CI
Hypertension	−1.227	0.625	3.849	0.050	0.293	(0.086 0.999)
Diabetes mellitus	−0.356	0.643	0.307	0.580	0.701	(0.199 2.468)
Chronic kidney disease	3.399	1.974	2.965	0.085	29.927	(0.625 1432.998)
BMI	0.085	0.096	0.784	0.376	1.089	(0.902 1.315)
ICU length of stay	0	0	4.380	0.036	1	(1.000 1.001)
EuroSCORE	−0.051	0.187	0.073	0.787	0.951	(0.659 1.372)
Preoperative WBC	0.137	0.116	1.397	0.237	1.146	(0.914 1.438)
Preoperative LAD	0.062	0.056	1.244	0.265	1.064	(0.954 1.187)
LVEF	0.013	0.028	0.232	0.630	1.014	(0.960 1.070)
Preoperative eGFR	−0.029	0.034	0.730	0.393	0.971	(0.908 1.039)
TyG Index	3.420	1.047	10.678	<0.001	30.573	(3.930 237.807)

BMI, body mass index; LVEF, left ventricular ejection fraction; WBC White blood cell count; LAD, left atrial diameter; eGFR, estimated glomerular filtration rate; TyG index, triglyceride-glucose index.

### 3.4 Tyg index for AKI

**Figure 2:**
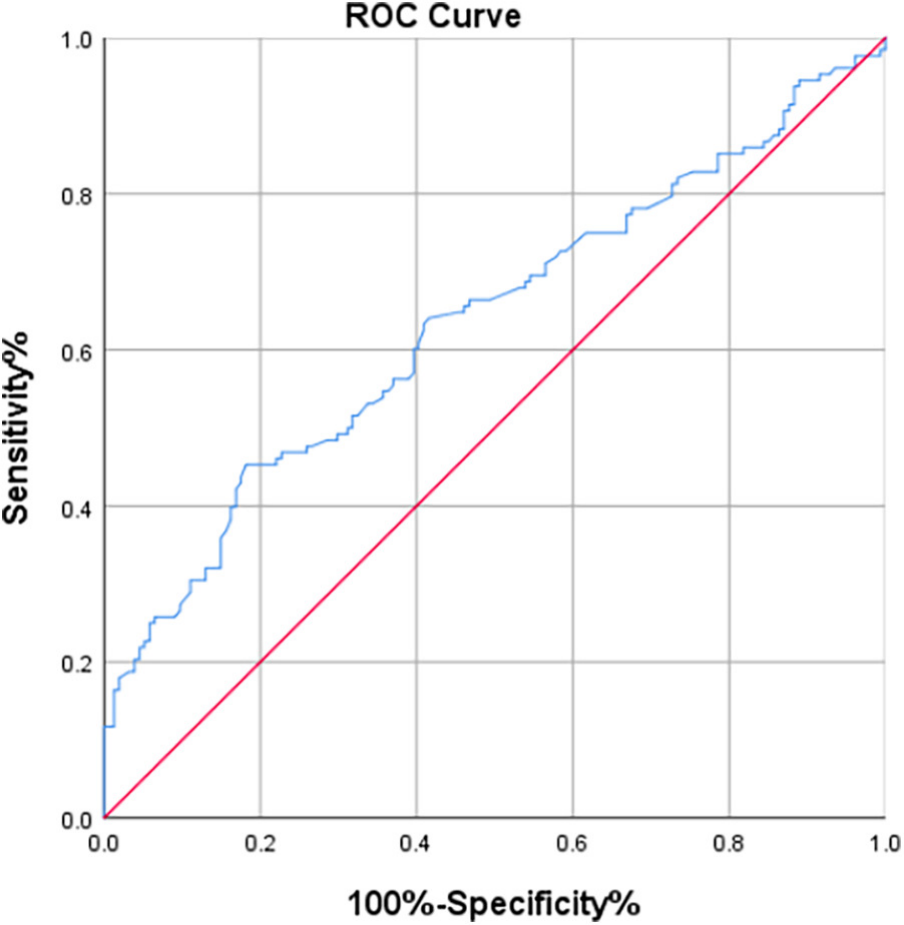
The ROC curve of TyG index for predicting AKI.

Further analysis involved the construction of a receiver operating characteristic (ROC) curve for the TyG index to predict the occurrence of AKI in patients after CABG surgery. The results indicated that the area under the ROC curve (AUC) for the TyG index in predicting postoperative AKI in CABG patients was 0.802 (*P* < 0.001; 95% CI 0.753–0.851). At a TyG index threshold of 7.20, the Youden's index was 0.506, Positive Predictive Value was 0.6815287, Negative Predictive Value was 0.8201439, with a sensitivity of 81.1% and a specificity of 69.5% (Figure 2). Compare the baseline characteristics of the two groups, and determine if there are statistically significant differences in gender, history of diabetes, Euroscore, preoperative total bilirubin, preoperative LVEF, preoperative fasting triglyceride levels, preoperative fasting blood glucose, and the incidence of AKI between the groups (*P* < 0.05, see Table 5).

**Table 5:** Comparison of preoperative baseline data and preoperative clinical indicators between two groups.

Overall population			
Variable	TyG index < 7.20 (*n* = 139)	TyG inde x≥ 7.20 (*n* = 157)	*P* value
Male, *n* (%)	116 (83.45)	115 (73.25)	0.036
Age (years)	67.00 [60.00, 73.00]	67.00 [57.00, 74.00]	0.588
BMI [median(IQR)]	23.80 ± 2.98	24.49 ± 3.08	0.092
NYHA, *n* (%)			0.764
I	25 (23.10)	26 (19.7)	
II	49 (45.40)	69 (52.3)	
III	32 (29.60)	35 (26.50)	
IV	2 (1.90)	2 (1.50)	
Hypertension, *n* (%)	104 (63.41)	99 (75)	0.078
Diabetes mellitus, *n* (%)	40 (28.78)	86 (54.78)	<0.001
CAD, *n* (%)	133 (95.68)	153 (97.45)	0.524
AF, *n* (%)	8 (5.76)	4 (2.55)	0.238
Hyperlipidemia, *n* (%)	10 (7.19)	14 (8.92)	0.672
MI, *n* (%)	13 (9.35)	20 (12.74)	0.460
Chronic lung disease, *n* (%)	10 (7.19)	12 (7.64)	1.000
Chronic kidney disease, *n* (%)	4 (2.88)	10 (6.37)	0.181
Euroscore	5.00 [4.00, 7.00]	5.00 [4.00, 7.00]	0.033
White blood cell count (x10^9^/L)	6.32 [5.17, 7.57]	6.54 [5.36, 7.98]	0.060
Neutrophil percentage (%)	61.70 [55.90, 67.20]	61.30 [53.70, 67.50]	0.647
Hemoglobin (g/L)	129.00 [118.00, 136.00]	127.54 ± 17.31	0.845
Platelet (x10^9^/L)	195.00 [164.50, 234.50]	200.00 [165.00, 238.00]	0.514
TBil (μmol/L)	11.60 [8.05, 14.40]	9.55 [7.57, 13.05]	0.014
ALB (g/L)	38.92 ± 3.15	39.52 [37.14, 41.92]	0.169
LAD (mm)	37.90 ± 5.40	38.00 [36.00, 41.00]	0.241
LVEDD (mm)	47.00 [44.00, 50.00]	47.00 [43.00, 51.00]	0.937
LVEF (%)	63.50 [56.00, 66.25]	60.00 [49.00, 65.00]	0.024
TG (mmol/L)	1.11 [0.89, 1.36]	1.88 [1.45, 2.41]	<0.001
FBG (mmol/L)	5.02 [4.50, 5.72]	6.87 [5.50, 8.80]	<0.001
Crea (μmol/L)	80.40 [69.15, 93.70]	84.60 [67.60, 101.00]	0.127
eGFR (ml/min)	77.57 ± 16.71	72.17 ± 21.70	0.199
AKI	25 (17.99)	107 (68.15)	<0.001

Data are expressed as the median (25th–75th percentiles), mean ± standard deviation, or number (percentage). BMI, body mass index; CAD, coronary heart disease; AF, atrial fibrillation; MI, myocardial infarction; TBiL, total bilirubin; Alb, albumin; LAD, left atrial diameter; LVEDD, left ventricular end-diastolic diameter; LVEF, left ventricular ejection fraction; TG, triglyceride;FBG, fasting blood-glucose; TyG index, triglyceride-glucose index; Crea, creatinine; eGFR, estimated glomerular filtration rate.

### 3.5 Calibration and clinical applicability analysis of the model

**Figure 3:**
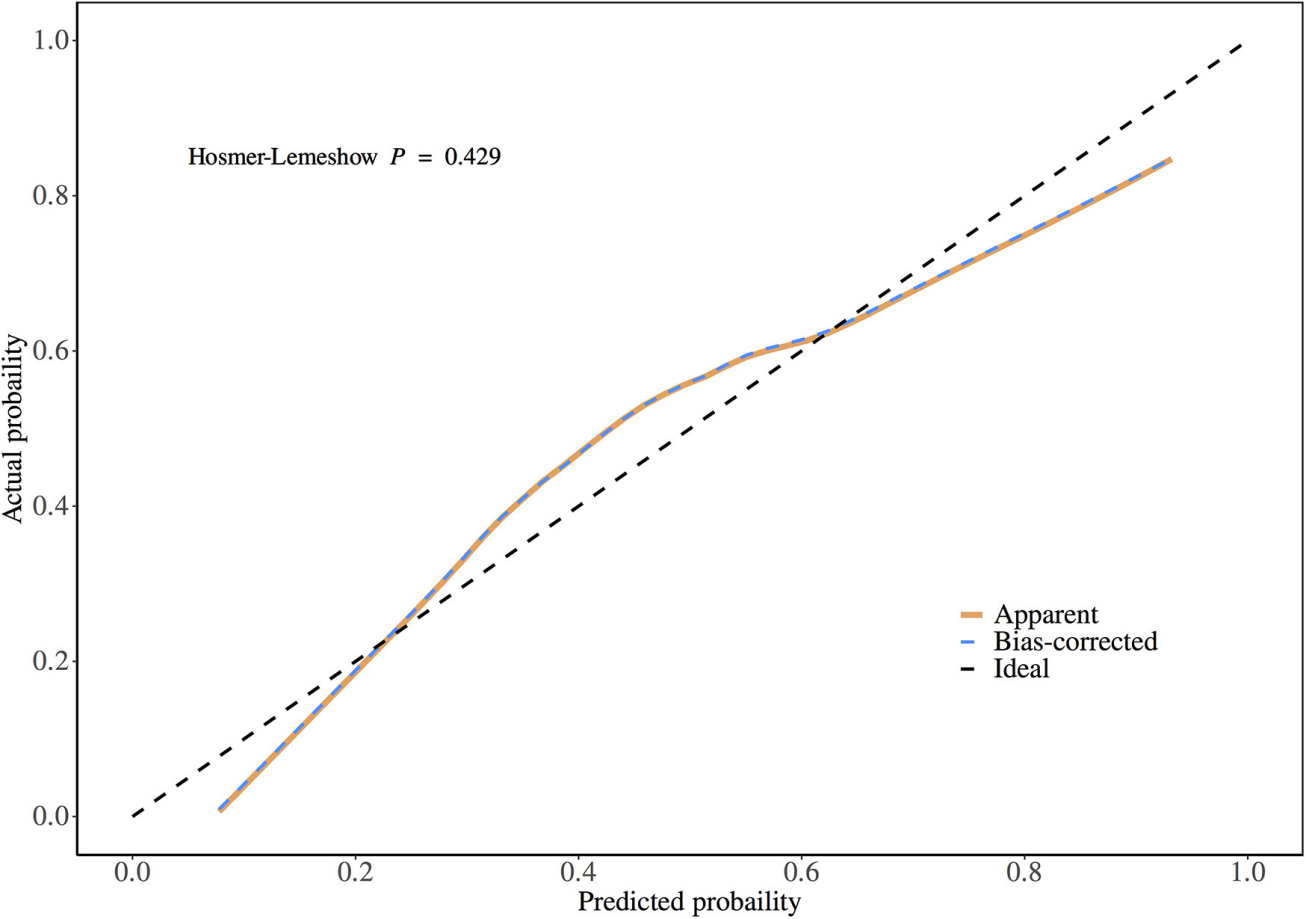
Calibration plot of the prediction model.

**Figure 4:**
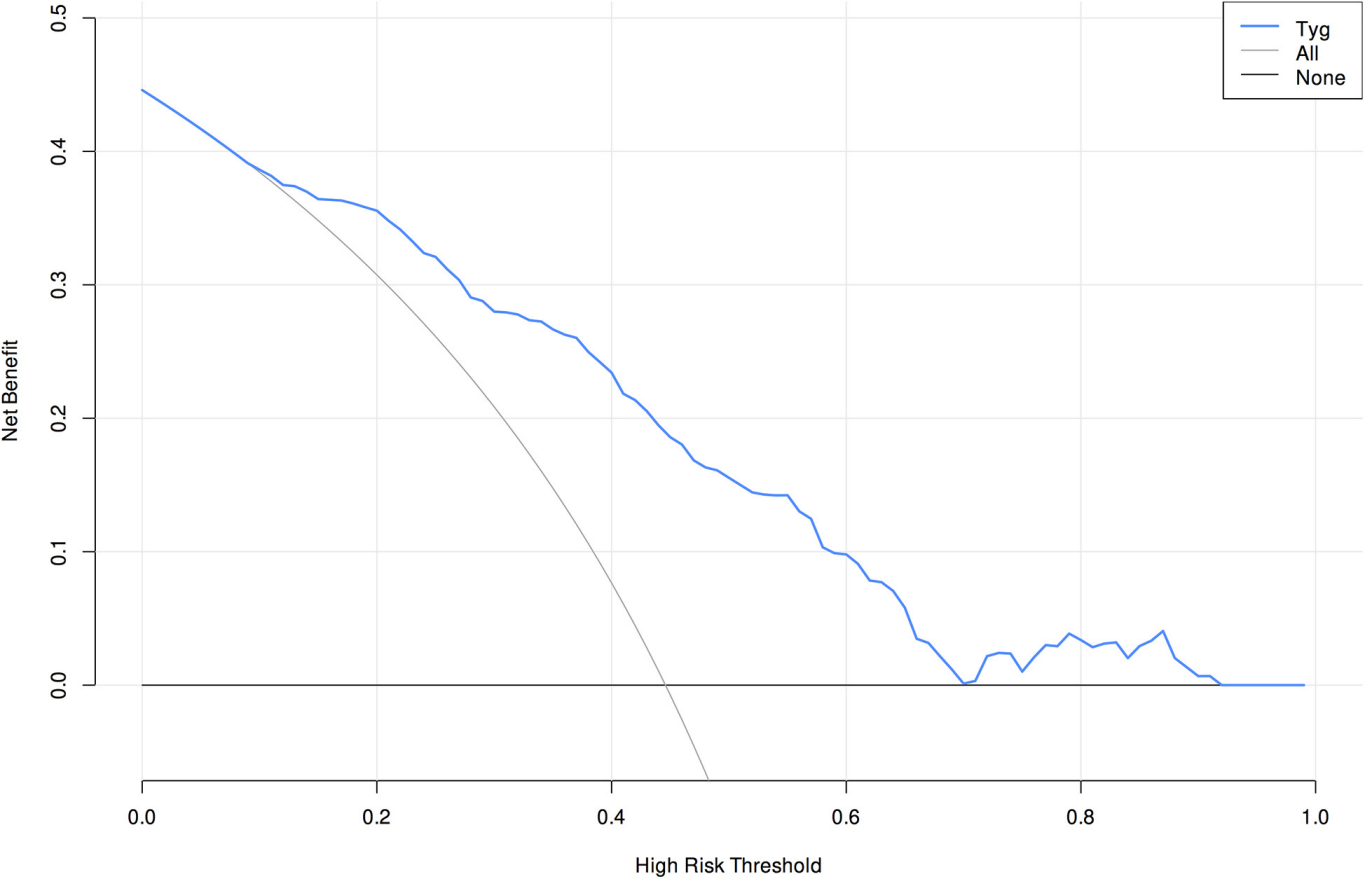
Decision curve analysis of the prediction model.

Validation of the TyG prediction model calibration was performed (Figure 3), with the calibrated curve closely aligning with the reference line, indicating good calibration performance. Decision curve analysis (DCA) was conducted (Figure 4), demonstrating a high net benefit when using this model to predict the occurrence of AKI in patients after CABG surgery at threshold probabilities ranging from 10% to 90%.

### 3.6 Line chart of creatinine changes

**Figure 5:**
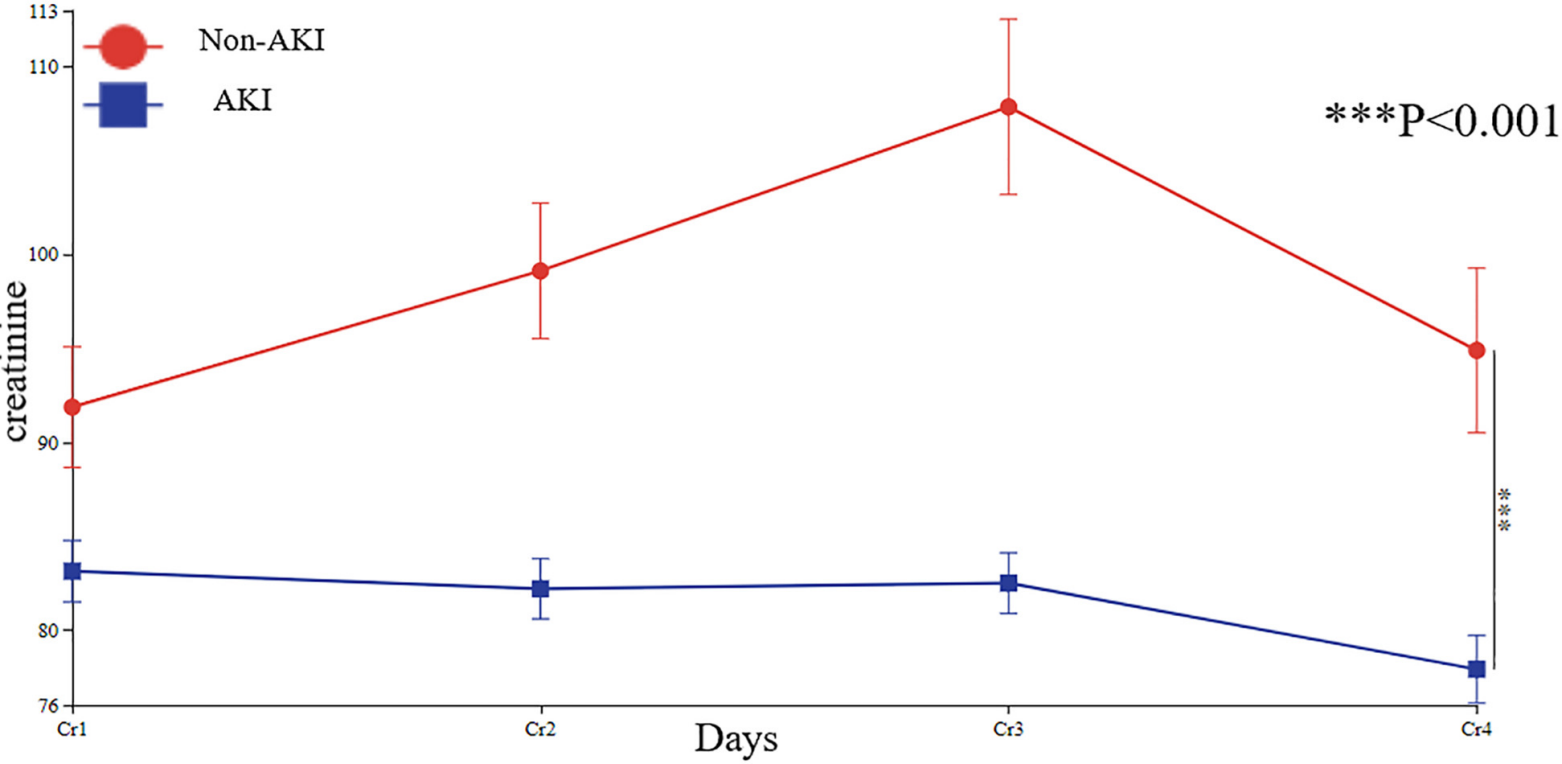
Line chart of patients' creatinine changes. Cr1, preoperative creatinine; Cr2, postoperative Day0 creatinine; Cr3, postoperative Day1 creatinine; Cr4, pre-discharge creatinine.

Compared to preoperative levels, creatinine exhibited an increasing trend postoperatively. However, pre-discharge creatinine levels showed a declining trend compared to postoperative values. This pattern was consistent in both the AKI and non-AKI groups, with pre-discharge creatinine levels decreasing relative to postoperative measurements in all patients (Figure 5).

### 3.7 Subgroup analysis

**Figure 6:**
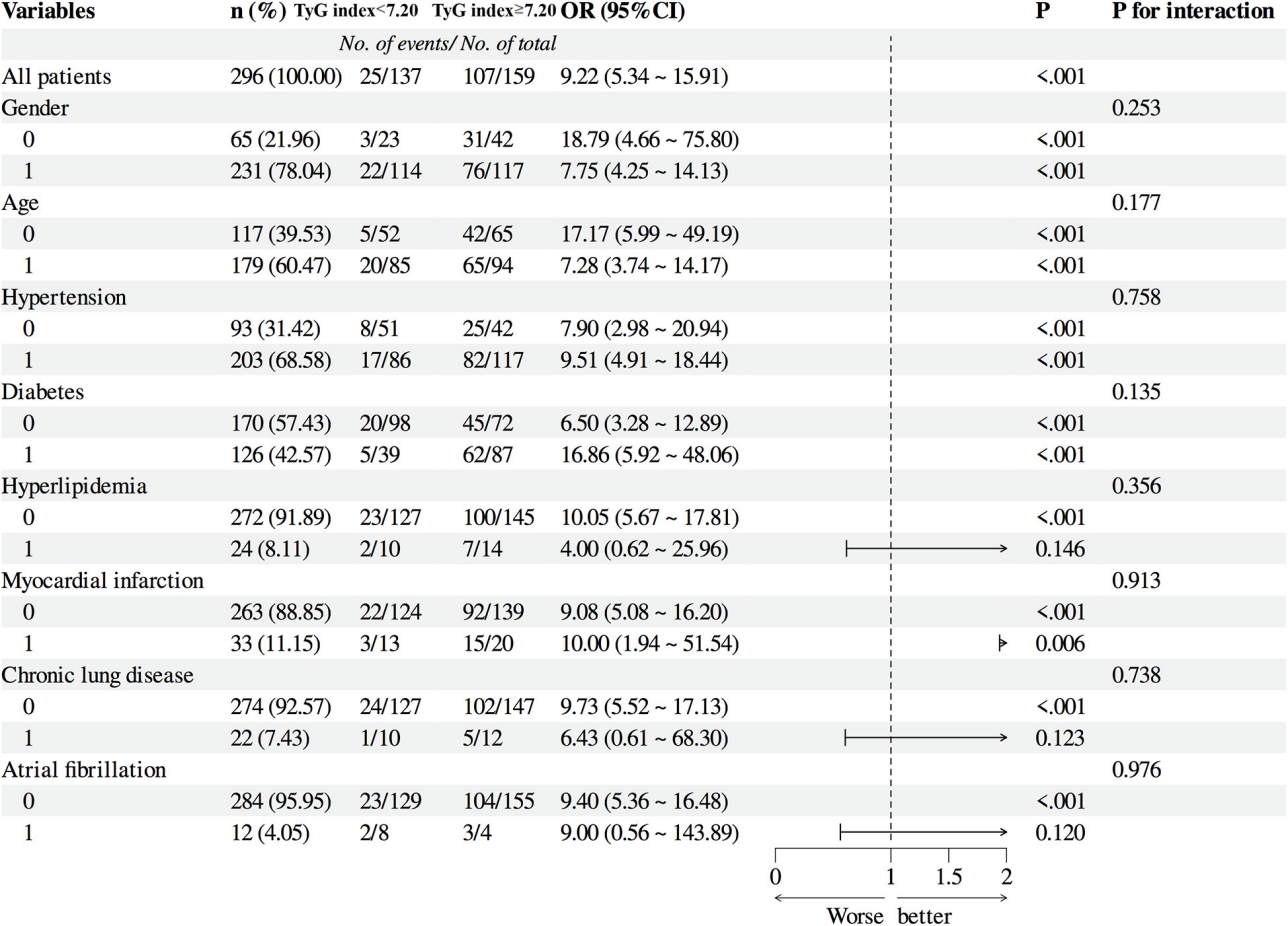
Subgroup Analysis of patients.

Subgroup analyses confirmed the prognostic value of the TyG index across diverse patient characteristics. When stratified by sex (groups 0 and 1), group 0 exhibited an event rate of 18.79 (95% CI: 4.66–75.80, *P* < 0.001), whereas group 1 showed an event rate of 7.75 (95% CI: 4.25–14.13, *P* < 0.001), with an interaction *P*-value of 0.253, indicating no significant sex-based effect. For age stratification (groups 0 and 1), group 0 had an event rate of 17.17 (95% CI: 5.99–49.19, *P* < 0.001), and group 1 demonstrated an event rate of 7.28 (95% CI: 3.74–14.17, *P* < 0.001), with an interaction *P*-value of 0.177, suggesting no age-related modification. In hypertension subgroups, non-hypertensive patients had an event rate of 7.90 (95% CI: 2.98–20.94, *P* < 0.001), compared to 9.51 (95% CI: 4.91–18.44, *P* < 0.001) in hypertensive patients, with an interaction *P*-value of 0.758. For diabetes stratification, non-diabetic patients exhibited an event rate of 6.50 (95% CI: 3.28–12.89, *P* < 0.001), whereas diabetic patients showed a significantly higher rate of 16.86 (95% CI: 5.92–48.06, *P* < 0.001), though the interaction *P*-value was 0.135. In hyperlipidemia subgroups, non-hyperlipidemic patients had an event rate of 9.51 (95% CI: 4.91–18.44, *P* < 0.001), vs. 4.00 (95% CI: 0.62–25.96, *P* = 0.146) in hyperlipidemic patients, with an interaction *P*-value of 0.356. Myocardial infarction (MI) subgroups showed event rates of 9.08 (95% CI: 5.08–16.20, *P* < 0.001) for non-MI patients and 10.00 (95% CI: 1.94–51.54, *P* = 0.006) for MI patients (interaction *P* = 0.913). For chronic pulmonary disease, non-affected patients had an event rate of 9.73 (95% CI: 5.52–17.13, *P* < 0.001), compared to 6.43 (95% CI: 0.61–68.30, *P* = 0.123) in affected patients (interaction *P* = 0.738). Lastly, in atrial fibrillation subgroups, non-affected patients exhibited an event rate of 9.40 (95% CI: 5.36–16.48, *P* < 0.001), vs. 9.00 (95% CI: 0.56–143.89, *P* = 0.120) in affected patients (interaction *P* = 0.976) (Figure 6).

## 4 Discussion

This study explored the relationship between the TyG index and the occurrence of AKI in patients after CABG surgery. The results of the multiple linear regression analysis indicated that the level of the TyG index is a significant influencing factor on the risk of AKI. Logistic regression analysis showed that patients with an elevated TyG index had a higher relative risk of renal dysfunction, suggesting an elevated TyG index having a predictive role for early renal injury in patients following off-pump CABG.

Insulin resistance, which is a condition affecting the body's overall ability to process glucose and lipids, is defined by the presence of elevated insulin levels (hyperinsulinemia), high blood sugar (hyperglycemia), and increased blood lipids (hyperlipidemia). Research has indicated that high blood sugar levels independently forecast the risk of developing AKI (14). Hyperglycemia can lead to disruptions in the kidney's blood flow and the permeability of its blood vessels. This can result in a series of complications such as the obstruction of capillaries, hypoxia, and glomerulosclerosis. These changes can contribute to the leakage of proteins into the urine (proteinuria) and potentially trigger AKI (15, 16). Additionally, dyslipidemia has been proven to be crucial in the development and progression of kidney disease (17). Although the mechanisms by which lipids damage renal vasculature, mesangial cells, and tubular cells are not yet fully understood, Moorhead first proposed the “lipotoxicity hypothesis” (18). Current research indicates that hypertriglyceridemia is an independent risk factor for early AKI (19).

Insulin resistance is a pivotal determinant in the etiology of cardiovascular diseases, diabetes mellitus, and renal impairment. The hormone insulin, through its distinct regulatory influence on metabolic and proliferative pathways within the kidney, modulates renal microcirculatory dynamics. This intricate interplay is essential for the kidney's homeostatic balance and its ability to manage fluid and electrolyte equilibrium (20). Insulin resistance often impairs renal blood flow and glomerular filtration, which may lead to issues such as inflammation and fibrosis (4, 20). Insulin resistance is a common comorbidity in individuals presenting with renal impairment, irrespective of their diabetic status. It is imperative to acknowledge that insulin resistance stands as a significant and autonomous predictor of renal pathology, underscoring its relevance in the pathogenesis of kidney disorders (21, 22). The euglycemic clamp technique is considered the criterion standard for evaluating insulin resistance, providing a measure of glucose metabolism rate via intravenous glucose infusion. Despite its accuracy, the technique's invasiveness and the associated economic burden constrain its routine use in clinical settings (23). The TyG index, a surrogate marker derived from fasting triglycerides and glucose, is widely used to assess insulin resistance. However, it is not a direct measure like the gold-standard hyperinsulinemic euglycemic clamp. The TyG index may be influenced by other metabolic factors, such as inflammation and fat distribution. Despite these limitations, it is valuable in large-scale studies and clinical practice due to its simplicity. In this study, it was observed that patients who developed AKI postoperatively had higher BMI values and a higher proportion of past medical histories of hypertension, diabetes, and chronic kidney disease, which is consistent with the findings of the aforementioned studies indicating that such patients are more prone to insulin resistance.

The TyG index, derived from fasting blood glucose and fasting triglycerides, is a reliable surrogate marker for insulin resistance. The TyG index has gained recognition as a pivotal prognostic marker for diabetes and atherosclerosis. Additionally, it demonstrates a strong correlation with proteinuria and the deterioration of renal function, highlighting its utility in the clinical assessment of metabolic and renal health (11, 24). Studies on renal impairment in elderly patients have demonstrated a positive and independent relationship between the TyG index and the deterioration of kidney function (25).

Studies have reported that the TyG index can effectively predict the onset and progression of diabetic nephropathy and chronic kidney disease. The kidney, being among the organs that are responsive to insulin, houses an array of cells that are sensitive to this hormone and exerts a distinctive function within the renal system. These functions include the regulation of glomerular filtration rate, the preservation of tubular sodium equilibrium, and the modulation of gluconeogenesis, thereby underscoring the kidney's integral role in both metabolic and excretory processes. If insulin resistance occurs, the insulin signaling pathway is impaired, leading to renal damage (4, 20, 26).

AKI is one of the most common postoperative complications in patients undergoing cardiac surgery. Additionally, AKI has an adverse impact on patient prognosis (27). According to various studies, the incidence rate ranges from 15% to 30%, depending on the type of surgery (28). Among these patients with AKI, approximately 2% to 5% require hemodialysis treatment, which significantly affects the morbidity, mortality, and hospitalization costs for surgical patients (28). It is important to recognize that AKI in the context of cardiac surgery is a multifaceted event that can manifest at various stages. It may arise during the preoperative preparation phase, continue to occur intraoperatively, and persist into the postoperative recovery period. This underscores the need for vigilant monitoring and proactive management of renal function throughout the entire perioperative course (29). AKI related to cardiac surgery leads to prolonged hospital stays, increased hospitalization costs, and a higher mortality rate. Early diagnosis and guidance for the protection of renal function are crucial. Historically, renal health has been gauged primarily through biochemical indicators such as serum creatinine and blood urea nitrogen. However, these markers have limitations in their sensitivity to detecting acute or short-term fluctuations in kidney function. Their delayed response to renal impairment makes them less than ideal for early detection of changes, particularly in settings where rapid assessment is crucial, such as in intensive care or following major surgery (30).

The increase in creatinine levels can only be observed several days after renal injury, which may lead to an underestimation of the incidence of acute kidney injury (31). Markers such as Neutrophil Gelatinase-Associated Lipocalin (NGAL) and Kidney Injury Molecule-1 (KIM-1) have drawbacks including low sensitivity and poor specificity, which prevent their widespread use in clinical practice (32, 33). Therefore, the relationship between the TyG index and AKI following cardiac surgery is imperative to explore. Previous studies have reported that the TyG index is significantly associated with a decrease in eGFR and diabetic nephropathy. However, the relationship between the TyG index and AKI after cardiac surgery has not yet been investigated. This study shows that patients who developed AKI postoperatively had a longer ICU stay, and both postoperative creatinine levels and eGFR were worse to varying degrees compared to preoperative levels, similar to the results of previous studies. The TyG index in patients who developed AKI after CABG surgery was significantly higher than in those who did not, indicating that the TyG index is a risk factor for the occurrence of postoperative acute kidney injury. The results of the ROC curve analysis suggest that the TyG index has some value in predicting the occurrence of acute kidney injury in patients after CABG surgery. The calibration plot demonstrated good predictive accuracy, and the clinical decision curve analysis (DCA) indicated favorable clinical applicability.

This study categorized patients into two groups based on the cut-off value from the ROC curve: one with a TyG index less than 7.20 and the other with a TyG index greater than 7.20. It was observed that there were more female patients in the group with a higher TyG index, and these patients were more likely to have comorbidities such as diabetes, hyperlipidemia, and hyperglycemia preoperatively. Assessments using the Euroscore and LVEF indicated that patients with higher TyG indices also had relatively higher surgical risks and poorer preoperative left cardiac function. Patients with elevated TyG indices had lower preoperative total bilirubin levels, which may be due to the association of high TyG index with non-alcoholic fatty liver disease, showing a negative correlation with bilirubin (34). Elevated levels of the TyG are significantly correlated with an increased propensity for the progression of non-alcoholic fatty liver disease (NAFLD) and a concomitant reduction in the likelihood of its remission. Elevation of serum total bilirubin has been found to be negatively correlated with the prevalence of NAFLD (35). Additionally, the risk of developing AKI was higher in patients with a high TyG index.

Based on the above data, the TyG index appears to be a highly reliable and effective indicator for predicting the incidence of AKI. Subgroup analyses showed that, though numerical risk ratio variations existed across subgroups, patient characteristics did not alter the TyG index-outcome association. The TyG index maintained consistent predictive accuracy in all subgroups, supporting its reliability as an AKI risk stratification tool independent of patient features. Our study results indicate a close correlation between elevated TyG levels and an increased incidence of AKI. AKI can ultimately lead to chronic kidney disease, uremia, or death. Our findings can assist in the clinical identification of high-risk patients and in taking preventive measures to avert the development of AKI. To date, clinical studies on the relationship between AKI and the TyG marker in patients following cardiac surgery have been limited. Our investigation fills this gap.

## 5 Limitations

This study has certain limitations. Firstly, the results were obtained from a single center, retrospective study, and selection bias is inevitable. This study was limited by a relatively small sample size, which may result in insufficient statistical power. Secondly, we only measured fasting triglycerides and fasting blood glucose upon admission, and these parameters were not continuously measured. This is a retrospective study, and due to incomplete 24 h urine output data, the diagnosis of acute kidney injury primarily relied on serum creatinine levels, potentially omitting patients with AKI identified by reduced urine output. Thirdly, we were only able to demonstrate a correlation between the TyG index and early renal injury in patients following off-pump CABG surgery, but the existence of a causal relationship would require further prospective studies to confirm.

## 6 Conclusion

The results of this study indicate that the TyG index is positively correlated with the occurrence of AKI following cardiac surgery. Elevated TyG levels are closely associated with an increased incidence of AKI, suggesting that the TyG index may be a valuable indicator for assessing the risk of AKI in patients after cardiac surgery. This finding offers new possibilities for the clinical identification of high-risk patient groups, and for the prevention and early intervention of AKI. However, further research is needed to validate the accuracy and reliability of the TyG index in predicting AKI after cardiac surgery.

## Data Availability

The raw data supporting the conclusions of this article will be made available by the authors, without undue reservation.
